# Quality Assessment of *Panax notoginseng* from Different Regions through the Analysis of Marker Chemicals, Biological Potency and Ecological Factors

**DOI:** 10.1371/journal.pone.0164384

**Published:** 2016-10-10

**Authors:** Hai-zhu Zhang, Da-hui Liu, Ding-kun Zhang, Yan-hui Wang, Gang Li, Gui-lin Yan, Li-juan Cao, Xiao-he Xiao, Lu-qi Huang, Jia-bo Wang

**Affiliations:** 1 Chengdu University of Traditional Chinese Medicine, Chengdu, 611137, China; 2 China Military Institute of Chinese Medicine, 302 Military Hospital, Beijing, 100039, China; 3 Dali University, Dali, 671003, China; 4 Yunnan Genuine Medicinal Materials Research and Development Centre, Kunming University of Science and Technology, Kunming, 650500, China; 5 China Medico Corporation, International building 811#, Guang-qu Men street, 80#, Dong-cheng district, Beijing, 100062, China; 6 China Academy of Chinese Medical Sciences, National Resource Centre for Chinese Materia Medica, Beijing, 100700, China; Chinese Academy of Medical Sciences and Peking Union Medical College, CHINA

## Abstract

*Panax notoginseng* (Burk.) F.H. Chen, called Sanqi in China, is a perennial herb that has been used as a medicinal herb in traditional Chinese medicine for more than 400 years. Because *notoginseng* is included in many proprietary Chinese medicines, the quality of *notoginseng* directly affects its efficacy and safety. However, considering the complex and special growth environment requirements of *notoginseng*, it is insufficient to evaluate its quality based solely on the analysis of marker chemicals. Thus, in this study, we tried to evaluate the quality of *notoginseng* with integrated indicators: (1) the concentration of five marker chemicals, notoginsenoside R1, ginsenoside Rg1, ginsenoside Re, ginsenoside Rb1 and ginsenoside Rd; (2) the anticoagulant activity (ACA); and (3) twenty-one ecological factors (e.g., longitude, latitude, elevation and soil data). Using these 27 parameters, *notoginseng* from different regions could be distinguished effectively, indicating a remarkable divergence of quality. A correlation analysis showed that variations of the ecological factors were closely associated with the saponins content and biopotency. For instance, the total nitrogen (TN), alkali hydrolysis nitrogen (AHN) and rapidly available potassium (RAPT) were significantly correlated with ACA, and RAPT was significantly correlated with the content of ginsenoside Rd and notoginsenoside R1. The results demonstrated that the high-quality *notoginseng* was produced from the emerging regions such as Kunming, Qujing and Honghe, which had higher ACA and saponin content than the *notoginseng* produced in traditional regions such as Wenshan and Baise.

## 1. Introduction

*Panax notoginseng* (Burk.) F.H. Chen, called sanqi in China, is a perennial herb that has been used as a medicinal herb in traditional Chinese medicine for more than 400 years[1–2]. *Panax notoginseng* belongs to the same genus as Chinese and Korean ginseng (*Panaxginseng*) and American ginseng (*Panax quinquefolium*) [3–4].

Extensive phytochemical and pharmacological studies on this plant proved the dammarane-type saponins to be the main bioactive principles [5–10], which are composed of a protopanaxadiol and protopanaxatriol glycosides [11–14].

Currently, *notoginseng* is a commonly used for treating cardiovascular diseases, such as lowering blood lipids, improving myocardial relaxation function, protecting arterial endothelium from injury, blocking Ca^2+^ influx into VSMCs, and oestrogen-like activity[15–23]. *Notoginseng* is also used for inflammation, anti-inflammation in atherosclerotic lesion of the aorta, body trauma, pain, and internal and external bleeding due to injury [24–29].

Because it has efficacy and lower adverse effects, *notoginseng* is gaining attention in Europe and America. At present, the State Food and Drug Administration (SFDA) approved production included 750 *Panax notoginseng* and *Panax notoginseng* drugs. Many proprietary Chinese medicines contain *notoginseng*, including *notoginseng* tablets, notoginseng injury tablets, compound *Panax notoginseng*, *notoginseng* tongshu capsules, and *notoginseng yang*xue capsules. In recent years, the number of traditional medicines using *notoginseng* has been increasing. Therefore, the quality of *notoginseng* medicinal materials is significant and will directly affect the safety and efficacy in clinic. However, the quality of *notoginseng* is influenced by several factors. The biological characteristics of *notoginseng* are determined by the special growth environment and the growth cycle. The growing conditions require warm weather in winter and cool weather in summer. The requirements for cold and heat, and moisture, are satisfied mostly at low latitude and high elevation areas, so specific *notoginseng* is found in the Wenshan Yunnan and Baise Guangxi geographic ranges. Among the two regions, Wenshan, where the drug yield exceeds 90% of the total, has been nominated as ‘‘Sanqi Hometown” [30,31]. As a result of the high medicinal value, the cultivated area in Wenshan and Baise[32], which are the traditional regions, has continuously increased. However, the cultivation of notoginseng demands specific soil, climate and geographical environment and more time than other herbs. Prominent problems due to the successive cropping obstacles in the traditional regions are increasingly, such as plant insect pests, soil rot, and the changing of nutrient, physical properties and microflora in the soil [33,34], will significantly impact the quality of *notoginseng*.

However, there is no comprehensive method to evaluate the quality. The current quality evaluation method measures the chemical composition, but there are numerous chemical elements in the herb that are unknow or cannot be determined. When the contents of the chemical components in different samples are the same, the biological activity can be significantly different between samples[35], so there other components at work. Considering the complex and special growth environment properties of *notoginseng*, and determining the chemical composition cannot reflect the quality objectively and comprehensively, therefore, it is essential to explore integrated evaluation methods.

In this study, we analysed the content of notoginsenoside R1, ginsenoside Rg1, ginsenoside Re, ginsenoside Rb1, and ginsenoside Rd; calculated the anticoagulant activity (ACA); analysed ecological factors, including longitude, latitude and elevation; and measured the pH, moisture, organic content, total nitrogen (TN), alkali hydrolysis nitrogen (AHN), nitrate nitrogen (NN), ammonia nitrogen (AN), total phosphorus (TP), rapidly available phosphorus (RAP), total potassium (TPT), rapidly available potassium (RAPT), commutativity calcium (CCa), commutativity magnesium (CMg), available iron (AFe), available manganese (AMn), available copper (ACu), available zinc (AZn), and available boron (AB) in soil. We sought to analyse the correlation between the producing area and the chemical constituents; analyse the biological activities and ecological factors; comprehensively compared the quality characteristics of *notoginseng* to explain the relationship between the chemical component content, biological activity and ecological factors, to determine the decisive factors that affect the quality of *notoginseng*; and provide integrated quality evaluation and a foundation for further search on the choice of a suitable location to grow *notoginseng*, to guarantee its quality and clinical curative effect.

## 2. Experimental

### Plant materials

The samples of *Panax notoginseng* were collected from Guangxi and Yunnan Province in China, cultivated three years (Table 1). The study was carried out on private land, we confirm that the owner of the land gave permission to conduct the study on this site and the field studies did not involve endangered or protected species.

**Table 1:** *Panax notoginseng* analysed in this study.

Sample No.	Source	East longitude(^。^)	Northern latitude(^。^)	Elevation(m)	Colletion year
1	Jingxi,Baise,Guangxi,	106.32	23.13	762	2014
2	Yanshan,Wenshan,Yunnan,	104.3	23.65	1603	2014
3	Qiubei,Wenshan,Yunnan,	104.17	23.98	1481	2014
4	Maguan,Wenshan,Yunnan,	104.45	22.95	1443	2014
5	Wenshan,Wenshan,Yunnan	104.23	23.43	1597	2014
6	Mile,Honghe,Yunnan	103.25	24.43	2091	2014
7	Guandu,Kunming,Yunnan	102.97	25.18	1986	2014
8	Xundian,Kunming,Yunnan	103.33	25.73	2114	2014
9	Yiliang,Kunming,Yunnan	104.00	24.88	1881	2014
10	Shizong,Qujing,Yunnan	103.07	24.57	1973	2014

All of the herbal samples were authenticated by Professor Xiaohe Xiao, and the voucher specimens were deposited in People's Liberation Army 302 Hospital, Army Institute of Traditional Chinese Medicine (TCM), Beijing, China.

### Chromatographic experiments

The chromatographic experiments were performed on an Agilent 1120 series HPLC system (Agilent Technologies Inc., Shanghai, China). The separation was conducted at 30°C on an Agilent Zorbax eclipse Shim-pack PREP-ODS(H) kit column (4.6 × 250 mm, 5 μm). The mobilephase consisted of solvent A (acetonitrile) and solvent B (water) flowing at 1 mL·min^−1^. The initial conditions were 18% A for 12 min, and a linear gradient was performed to increase from 18% A to 38% A within 23 min, and then 38% A within 7 min, which was held for 0.5 min before returning to 18% A. The scan range for PDA was 203 nm with a sample size 20 μL.

The five reference compounds, notoginsenoside R_1_ (110745–200617), ginsenoside Rg_1_ (110703–201027), ginsenoside Rb_1_ (110704–201122), ginsenoside Rd (111818–201001) and ginsenoside Re (110754–200822), were accurately weighed: 1mg was dissolved in a 10 mL volumetric flask with methanol to produce standard stock solutions. Samples of herbal materials were ground into a fine powder and then passed through a 20 mesh (0.9 mm) sieve. Sample powder (0.3 g) was accurately weighed and transferred into a 50 mL triangle flask. Then, 100% methanol (25 mL) was added, the samples were stored over- night, and were then ultrasonicated for 30 min. When cool, methanol was added to compensate for weight loss. After filtering through a 0.45μm filter membrane, the filtrate was ready to be used.

### Biopotency assay experiments

The standard compound, aspirin, was accurately weighed: 50 mg was dissolved in a 10 mL volumetric flask with physiological saline to produce standard stock solutions, and the defined biological value was 500U·g^-1^. Samples of herbal materials were ground into fine powder and passed through a 20 mesh (0.9 mm) sieve. Sample powder (10 g) was accurately weighed and transferred into a 250 mL triangle flask. Deionized water (100 mL) was added, and the samples were allowed to sit for 30 min, before being ultrasonicated twice for 30 min. Samples were then merged, enriched, decompression dried, and prepared with normal saline solution to produce sample stock solutions at 15mg·mL^-1^. Ear marginal blood was collected by needle from two New Zealand rabbits (Males 2.5–4 kg) (SCXK 2011–0004), that were fasting 12 h. Blood was transferred into a sodium citrate anticoagulation tube, centrifuged for 15 min at 3000 r/min, and the plasma was stored at 4°C. The plasma and 200 μL of sample or standard stock solution were mixed at 37°C for 180 s, and the activated partial thromboplastin time (APTT) was detected by an automatic coagulation analyser (ACA)(Sysmex CA-7000, Japan). The experimental procedures and the animal use and care protocols were approved by the Committee on Ethical Use of Animals of 302 Military Hospital of China.

### Soil analysis

Soil samples were obtained from 0 to 15 cm soil layer. The surface mulch was removed, and the samples were placed in bags. The soil samples were dried in the shade to a constant weight under natural conditions and were passed through a 20 mesh and 100 mesh sieve.

pH was measured by the potentiometric method (water: soil = 2.5:1); the organic matter content was measured using the potassium dichromate volumetric method; total nitrogen (TN) was determined by Kjeldahl nitrogen distillation; alkali hydrolysis nitrogen (AHN, nitrate nitrogen (NN), and ammonia nitrogen (AN) were measured using the alkali solution diffusion method; total phosphorus (TP) was determined using the sodium hydroxide melting—molybdenum antimony colorimetric method; rapidly available phosphorus (RAP) was determined using the Olsen method; total potassium (TPT) was measure by sodium hydroxide melting—atomic absorption spectrophotometry; rapidly available potassium (RAPT) was measured using ammonium leaching acetic acid—atomic absorption spectrophotometry; changeable calcium (CCa) and magnesium (CMg) were determined using the EDTA—acetic acid ammonium exchange method; and available iron, manganese, copper, zinc, and boron (AFe, AMn, ACu, AZn, AB) were measured DTPA extraction—atomic absorption spectrophotometer.

## 3. Results and Discussion

### Analysis of the chemical compounds

**Fig 1:**
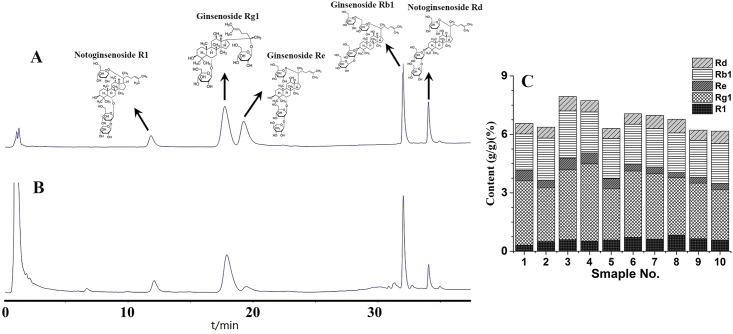
Chromatogram. (A) mixed standard solution. (B) sample solution. (C) content determination results.

The linearity, regression, and linear ranges of five investigated compounds were determined by HPLC. The data indicated a good relationship between the concentrations and peak areas of the compounds within the test ranges (*R*^*2*^≥ 0.9990). The LOQs and LODs of all compounds were less than 6.15 and 13.17μg·mL^−1^ (Table 2). The overall RSDs of the intra- and inter-day variations for analytes were not more than 2.11% and 11.54%, respectively. The established method also had acceptable accuracy, with spike recovery of 98.31–103.57% for all analytes. For the stability test, the RSDs of the peak areas for compounds detected within 12 h were lower than 2.59%. These results demonstrated that the HPLC method was linear, sensitive, precise, accurate, and stable enough for simultaneous quantification of the five investigated compounds in *Panax notoginseng*. Ten batches from different regions were analysed, as shown in Fig 1.

**Table 2:** Calibration curves, LODs, LOQ, and precision for notoginseng saponin R_1_, ginseng saponin Rg_1_, ginseng saponin Re, ginseng saponin Rb_1_ and ginseng saponin Rd.

Reference samples	Caliration curves	*R*^*2*^	Test ranges(mg·mL^−1^)	LODs(μg·mL^−1^)	LOQs(μg·mL^−1^)
Notoginseng saponin R_1_	*Y* = 1*E*+06*X*-1075.1	0.9999	0.018–0.146	5.01	11.25
Ginseng saponin Rg_1_	*Y* = 1*E*+06*X+*302.9	1	0.080–0.280	4.23	11.11
Ginseng saponin Re	*Y* = 1*E*+06*X*-2850.8	0.9995	0.020–0.180	6.15	12.22
Ginseng saponin Rb_1_	*Y* = 1*E*+06*X*-2187.4	0.9994	0.095–0.285	5.96	13.17
Ginseng saponin Rd	*Y* = 1*E*+06*X*-671.9	0.9999	0.033–0.415	5.17	12.15

### Analysis of the bioactivity of *Panax notoginseng*

**Fig 2:**
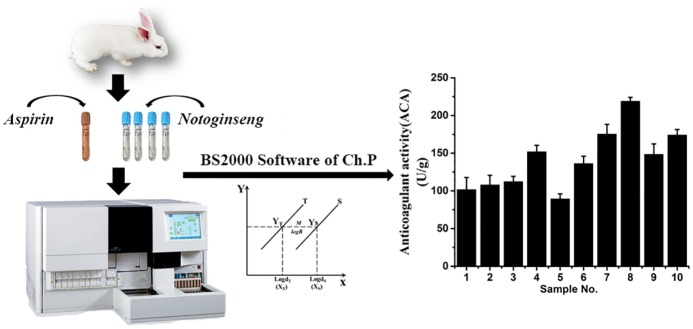
The result of the anticoagulant activity (ACA) analysis of *notoginseng*.

The analysis of the anticoagulant activity (ACA) used the quantum parallel reaction method of the State Pharmacopoeia Committee of Establishment of China Pharmacopoeia of Bioassay Statistical Program BS2000 technology. The biological reliability verification results are shown in Table 3 and Fig 2. The regression had a significant difference (*P* < 0.01), indicating that APTT increased regularly with the dose increase. The agent had a significant difference (*P* < 0.01), indicating that the test dose rate and test arrangement were reasonable. Deviation from parallel had no significant difference (*P* > 0.05), suggesting a parallel linear relationship between the standard group (S) and the test group (T). The ACA was from 89.47 to 218.87U ·g^−1^ in ten batches from different origin samples, and varied by nearly three times in different origin samples which indicated that the sample origin had a strong influence on the quality of *notoginseng* medicinal materials. For example, Xundian County in Kunming (sample No. 8), had the highest ACA and was Wenshan County in Wenshan (sample No. 5) had the lowest ACA.

**Table 3:** Results of the biological reliability verification.

Sources of variation	Degrees of freedom	Sum of squares of deviations	Variance	*F* value	*P* value
Between test samples	1	7.938	7.938	6.1967	>0.05
Regression	1	33.282	33.282	25.981	<0.01
Deviation from parallel	1	0.128	0.128	<1	>0.05
Between the agent	3	41.351	13.784	10.76	<0.01
Error	16	20.496	1.281		

### Analysis of soil characteristics

Fifteen soil characteristics, including TN, AHN, NN, AN, TP, RAP, TPT, RAPT, CCa, CMg, AFe, AMn, ACu, AZn, and AB in 10 batches of different regions of *notoginseng* were analysed as shown in Table 4. The producing region in Guandu County in Kunming (sample No. 7) had the highest AHN, TN, NN, and RAPT. N and K were the main nutrient elements of *notoginseng*. The application of N and K fertilizer was an important method to ensure the quality of *notoginseng* [36–38]. Kunming Region exhibited good quality soil owing to the high soil N and K content.

**Table 4:** Results of soil information verification.

Sample No.	pH	Moisture(%)	Organic (g·kg^-1^)	TN(g·kg^-1^)	ANH(mg·kg^-1^)	NN(mg·kg^-1^)	AN(mg·kg^-1^)	TP(g·kg^-1^)	RAP(mg·kg^-1^)	TPT(g·kg^-1^)	RAPT(mg·kg^-1^)	CCa(cmol·kg^-1^)	CMg(cmol·kg^-1^)	AFe(mg·kg^-1^)	AMn(mg·kg^-1^)	ACu(mg·kg^-1^)	AZn(mg·kg^-1^)	AB(mg·kg^-1^)
1	5.47	29.0605	33.3093	1.4023	136.0800	12.3951	1.3270	0.6736	3.7178	2.4610	121.87	4.9542	0.3034	70.90	30.18	6.931	3.7940	0.44
2	6.00	25.0064	29.7233	1.7840	110.3235	8.4634	2.3424	1.1641	30.2606	9.6935	230.02	7.4866	1.0851	86.15	275.40	4.234	6.5700	0.53
3	5.72	22.4473	20.5816	1.3358	86.5830	5.0594	2.2742	1.5564	27.1370	15.1522	252.75	4.8902	1.1880	72.06	265.02	5.032	4.7610	0.31
4	5.49	22.2327	18.0349	2.0959	126.6160	11.7949	1.9670	0.6911	8.9288	5.8668	236.75	4.6002	0.5220	111.75	75.80	3.231	6.3500	0.12
5	4.94	25.4633	26.7598	1.1255	97.2895	25.3621	2.3850	0.6141	5.4243	11.7107	117.90	3.0704	0.4911	14.97	16.54	0.453	3.1110	0.10
6	4.86	27.0974	21.2090	2.2668	158.2700	14.2671	4.2570	1.4338	26.1466	3.9659	174.22	2.7164	0.3394	42.58	185.63	7.013	4.4010	0.27
7	6.00	18.0471	46.0884	2.4226	202.9580	74.7563	1.9099	0.8256	18.1472	9.6816	418.42	7.2402	3.1397	61.95	234.01	4.344	6.6740	0.18
8	5.55	29.3345	32.4982	2.1573	183.8725	18.5088	2.0743	1.6053	19.8994	4.3182	310.32	3.7488	0.8511	67.88	201.32	9.711	7.0060	0.42
9	4.94	27.1017	36.7207	2.2235	184.3380	41.1349	1.5150	1.3553	29.6511	7.7961	275.97	2.4061	0.6943	40.32	101.62	2.216	3.0940	0.37
10	6.17	28.0474	41.8678	2.3010	143.3740	26.0160	3.1408	1.4496	59.0584	5.2267	363.27	5.9054	1.7897	61.30	251.81	5.304	9.1360	0.48

### The correlation analysis

**Fig 3:**
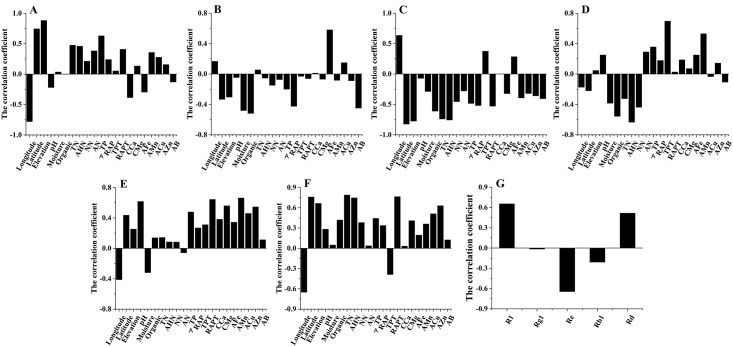
Correlation analysis. (A-E)The correlation between the content of chemical compounds (notoginsenoside R1, ginsenoside Rg1, ginsenoside Re, ginsenoside Rb1, ginsenoside Rd) and ecological factors. (F)The correlation between bioactivity and ecological factors. (G)The correlation between the content of chemical compounds and bioactivity.

**Fig 4:**
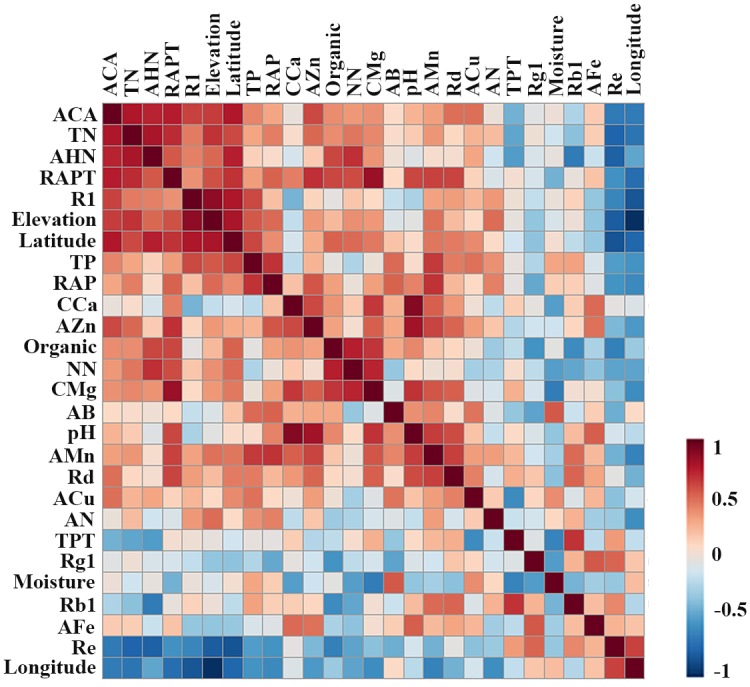
Thermograph of the correlation analysis of all factors.

**Fig 5:**
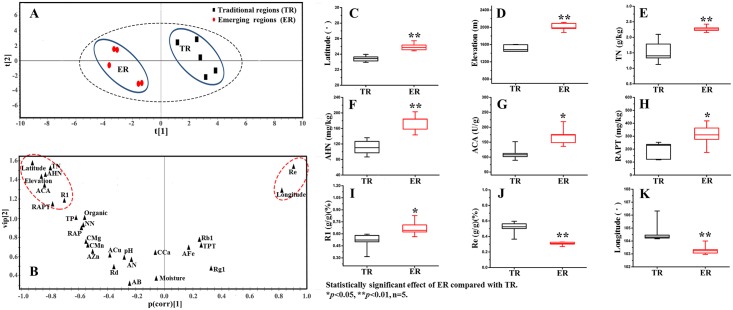
(A)PLS-DA of the producing regions versus the chemical constituents, biological activities and ecological factors. (B)The v-plot of the variables to distinguish between traditional regions (TR) and emerging regions (ER). (C-K) The decisive quality characteristics comparison of notoginseng from TR and ER.

From the data on the correlation coefficient among the main chemical compounds, and the bioactivity and ecological factors of *notoginseng* in Figs 3 and 4, we can see that notoginsenoside R1, ginsenoside Rd and anticoagulant activity(ACA) were significantly negatively correlated with the longitude and were significantly positively correlated with the latitude and elevation. Notoginsenoside R1 and ginsenoside Rd were significantly positively correlated with ACA (*P<*0.05), which indicates that the higher the content of notoginsenoside R1 and ginsenoside Rd, the higher the ACA. Notoginsenoside R1 was positively correlated with the TN, AHN, TP and RAPT. Ginsenoside Rd was positively correlated with AFe. Ginsenoside Rb1 was significantly positively correlated with TPT (*P<*0.05). Ginsenoside Rd was significantly positively correlated with AMn and RAPT (*P<*0.05). Ginsenoside Re was significantly negatively correlated with TN and AHN (*P<*0.05). The anticoagulant activity was significantly positively correlated with TN, ANH, RAPT and ginsenoside R1(*P<*0.05). Fig 4 presents the positive correlation relationship between the several factors, including ACA, TN, AHN, RAPT, R1, elevation and latitude. Re and longitude had a negative correlation relationship with the other factors. Fig 5 shows that the *notoginseng* samples were divided into 2 main clusters in the PLS-DA. The cultivated regions in Wenshan and Baise, which were the Sanqi hometown, were defined as the traditional regions (TR), and the regions in Kunming, Honghe and Qujing were defined as the emerging regions (ER). Such division indicated that different producing regions could significantly distinguish from TR to ER by the different chemical constituents, biological activities and ecological factors of *notoginseng*. The v-plot results of the variables to distinguish between TR and ER are shown in Fig 5. The ACA, latitude, elevation, longitude, TN, AHN, Re and longitude were the important factors in distinguishing between TR and ER. Fig 5 C-K shows an increasing tendency in latitude, elevation, TN, AHN, ACA, and RAPT, and a decreasing tendency in longitude and the content of Re from TR to ER. Therefore, the higher latitude, elevation, TN, AHN, ACA, and RAPT and lower of longitude and Re were the main reasons that the quality characteristics of TR cultivated *notoginseng* were worse than ER cultivation. The production of high-quality *notoginseng* has been migrating from TR, which has been cultivated hundreds years, to ER in recent decades. The prominent problems due to the successive cropping obstacles in the TR, such as plant insect pests, soil rot, and the changing of nutrient, physical properties and microflora in soil are increasing. The ER, has the superior specific soil, climate and geographical environment to TR, resulting in high-quality *notoginseng*.

## 4. Conclusions

The quality of *notoginseng* medicinal materials is not only affected by the chemical constituents. In this study, a reliable quality assessment through the analysis of marker chemicals, biological potency and ecological factors was established for *Panax notoginseng* from different regions. We found that high-quality *notoginseng* was produced by emerging regions such as Kunming, Qujing and Honghe, which had higher ACA and saponin content than the *notoginseng* produced in traditional regions such as Wenshan and Baise.

## Supporting Information

S1 File Approval of Experimental Animal Welfare and Ethics. (PDF)

S1 Table The results of component content determination. (DOC)
