# Supplementary material for: Quality Assessment of Panax notoginseng from Different Regions through the Analysis of Marker Chemicals, Biological Potency and Ecological Factors
Source: PLoS One. 2016 Oct 10;11(10):e0164384. doi: 10.1371/journal.pone.0164384 (PMC5056725; doi:10.1371/journal.pone.0164384)
Supplement: S1 File — (PDF) [file pone.0164384.s001.pdf]

Approval of Experimental Animal Welfare and Ethics

(302 Military Hospital)

Approval ID: IACUC-2016-056	Acceptance No:IACUC-2016-056	
Approval date: April 20,2016	Acceptance date: March 25,2016	
Applicant: Haizhu Zhang	Department:China Military Institute of Chinese Medicine	
Application date: March 24,2016	Certificate No: 2016021216	
Tel.: +86 10 66933325	Principal Investigator: Jia-bo Wang	
Fund: National Natural Science Foundation of China (81274026, 81403126)		
Review staff: Yan-ling Zhao, Zhao-fang Bai, Ya-ming Zhang, Hong-hui Shen, Rui-sheng Li		
Review mode	□Correspondence review	□Meeting review
Review decision	□Pass review	□Fail review
	Illustration: The experiment complies with the animal welfare and ethics and it is approved.	
Signature of the director: Rui sheng Li (中国人民解放军第三〇二医院 动物实验中心)	Date: April 20, 2016	
Additional remarks:		
