# Supplementary material for: Quality Assessment of Panax notoginseng from Different Regions through the Analysis of Marker Chemicals, Biological Potency and Ecological Factors
Source: PLoS One. 2016 Oct 10;11(10):e0164384. doi: 10.1371/journal.pone.0164384 (PMC5056725; doi:10.1371/journal.pone.0164384)
Supplement: S1 Table — (DOC) [file pone.0164384.s002.doc]

S1 Table. The results of component content determination

| Sample No. | Content of chemical component | | | | |
| --- | --- | --- | --- | --- | --- |
|  | ginsenoside R1 （g/g）(%) | ginsenoside Rg1（g/g）(%) | ginsenoside Re（g/g）(%) | ginsenoside Rb1（g/g）(%) | ginsenoside Rd （g/g）(%) |
| 1 | 0.3159 | 3.3219 | 0.5246 | 1.8650 | 0.5352 |
| 2 | 0.5001 | 2.7868 | 0.3652 | 2.1603 | 0.5586 |
| 3 | 0.5954 | 3.5831 | 0.5959 | 2.4349 | 0.7323 |
| 4 | 0.5230 | 3.9615 | 0.5630 | 2.1068 | 0.5744 |
| 5 | 0.5799 | 2.6446 | 0.5022 | 2.0693 | 0.5075 |
| 6 | 0.7115 | 3.4105 | 0.3257 | 2.0779 | 0.5285 |
| 7 | 0.6231 | 3.3523 | 0.3289 | 2.0005 | 0.6674 |
| 8 | 0.8275 | 2.9456 | 0.2694 | 2.0393 | 0.6974 |
| 9 | 0.6441 | 2.8561 | 0.3014 | 1.8981 | 0.5153 |
| 10 | 0.5643 | 2.6143 | 0.3111 | 2.0451 | 0.6304 |
